# Elbow arthroplasty complicated by *Mycobacterium tuberculosis* infection: A case report

**DOI:** 10.1097/MD.0000000000024376

**Published:** 2021-03-05

**Authors:** Yun Guan, Zhimin Zeng

**Affiliations:** a Medical Center; b Department of Orthopaedic Surgery, Ningbo No. 6 Hospital, Ningbo, Zhejiang, China.

**Keywords:** *Mycobacterium tuberculosis*, periprosthetic infection, rheumatoid arthritis, total elbow arthroplasty

## Abstract

**Introduction:**

Total elbow arthroplasty (TEA) is an orthopedic procedure that is relatively infrequently performed, but its use has been increasing over time. Infection remains one of the most concerning complications after TEA, although *Mycobacterium tuberculosis* (TB) as a microbial etiology, is extremely rare. Here, we present a case of *M. tuberculosis* infection after TEA.

**Patient concerns:**

A 45-year-old woman underwent TEA for severe traumatic arthritis of the elbow following failure of conservative treatment. Four months after TEA, the patient experienced progressive elbow pain and swelling, without other external signs of infection such as a sensation of local heating and erythematous alterations.

**Diagnosis:**

Pulmonary computed tomography showed stable pulmonary TB in the right upper lobe. The T-SPOT, TB, and purified protein derivative test results were positive, and *M. tuberculosis* exhibited growth on cultures. The final diagnosis was periprosthetic infection of *M. tuberculosis*.

**Interventions:**

The patient was treated with debridement with submission of deep tissue cultures. According to these cultures and suggestions of a bacteriologist, anti-TB treatment was administered for 12 months.

**Outcomes:**

The symptoms of the infection were controlled, and the prosthesis was retained. At the time of writing this case report, the elbow prosthesis had survived for more than 2 years, and no recurrent infection had been observed.

**Conclusion:**

The diagnosis of TB infection after TEA is difficult to confirm due to its nonspecific signs and symptoms. Despite the extremely low incidence, failure to consider this possibility for diagnosis can lead to delayed treatment. Proper diagnosis allows for antitubercular therapy with retention of a prosthesis.

## 1 Introduction

Total elbow arthroplasty (TEA) is an orthopedic procedure that is relatively infrequently performed compared to total knee or hip arthroplasty. According to the Dutch Arthroplasty Register in 2016, approximately 400 elbow arthroplasties were performed compared with 28,000 hip arthroplasties and 27,000 knee arthroplasties.^[1]^ For years, TEA has been performed mostly for patients with polyarticular inflammatory arthritis, but the indications have now expanded. Elbow arthroplasty is often considered for patients with severe unfixable distal humeral fractures or trauma sequelae.^[2]^ Patients undergoing this procedure may develop an infection after surgery, and the incidence of infection varies from 1% to 9%, which is seemingly higher than that in patients undergoing hip or knee arthroplasty.^[3]^ The most common infectious organisms are the Staphylococcus species, and infection with *Mycobacterium tuberculosis* is extremely rare. To the best of our knowledge, few reports of this uncommon complication exist in the literature, but failing to consider this type of infection as a possibility may lead to delayed treatment and poor patient outcomes. Here, we present a case of *M. tuberculosis* infection after TEA.

## 2 Case report

**Figure 1:**
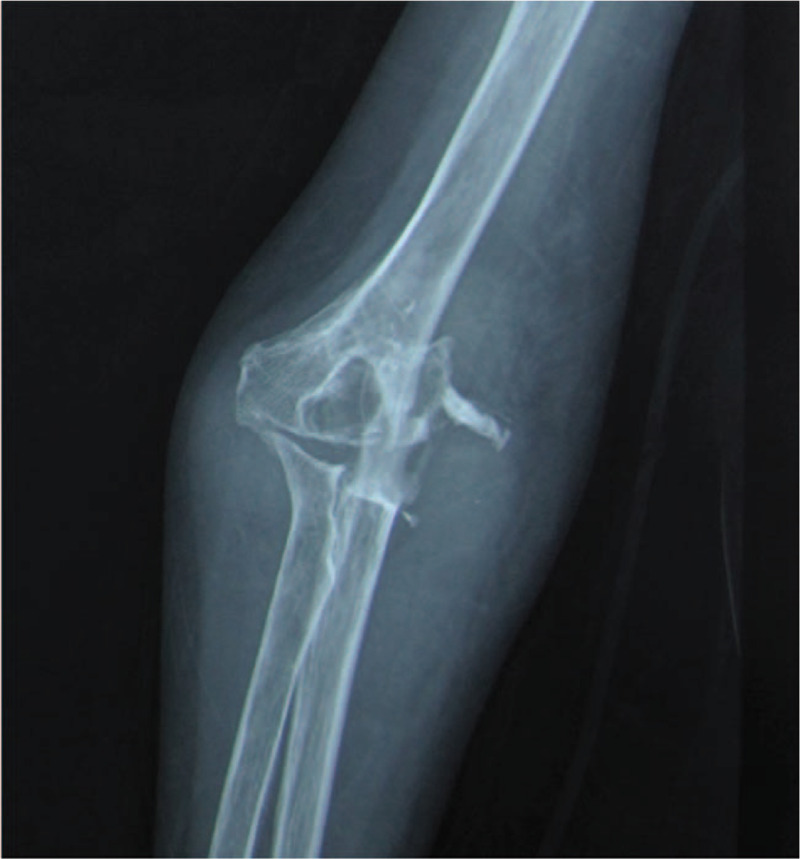
Anteroposterior view of the right elbow, showing severe osteoarthritis.

**Figure 2:**
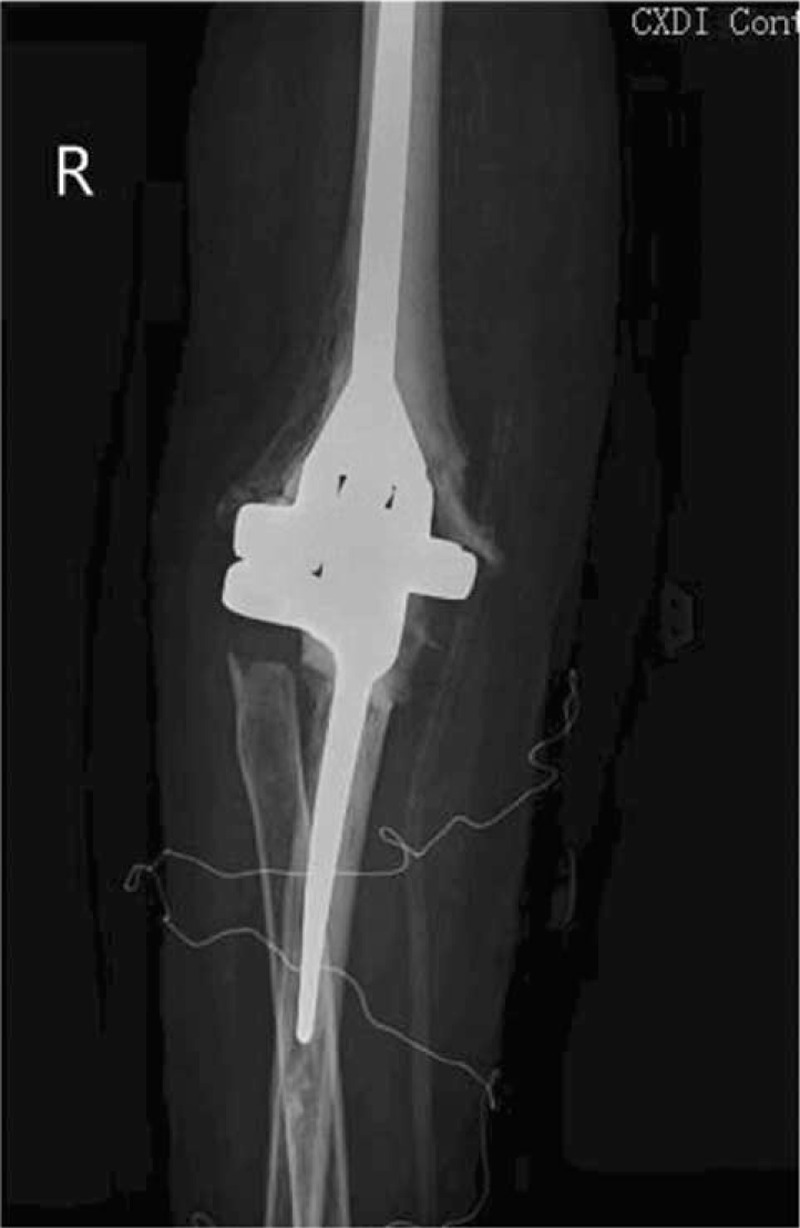
Anteroposterior view of the right elbow after TEA, showing good implant positioning. TEA = total elbow arthroplasty.

A 45-year-old woman was admitted to the hospital with severe osteoarthritis of the right elbow (Fig. 1). She complained of pain and stiffness in the bilateral elbow joint for 10 years, and the symptoms in the right elbow were aggravated due to a fall on the joint 3 years prior. She had a 10-year history of rheumatoid arthritis (RA) and was positive for treatment with tripterygium glycoside and methotrexate. After failure of nonoperative treatment, TEA was scheduled to be performed using a posterior approach. The preoperative diagnosis was RA with traumatic arthritis in the right elbow. Laboratory tests showed a normal erythrocyte sedimentation rate (ESR) and C-reactive protein (CRP) concentration before the surgery, and pulmonary computed tomography (CT) showed multiple microcalcifications in the right upper lobe. The surgery was performed successfully under general anesthesia, and cement-linked implants were employed for the patient. At the time of TEA, there was significant synovial hyperplasia and osteochondral destruction without caseous necrosis. We conducted a pathological examination of the synovial tissue, and the postoperative pathology was consistent with that of rheumatoid arthritis, culture of the synovial tissue was not performed. Postoperative plain radiography showed good implant positioning (Fig. 2). Flexion and extension of the elbow joint were encouraged on the first day after TEA, and she was discharged 4 days later.

Unfortunately, the patient presented to the clinic complaining of progressive pain and swelling in the right elbow 4 months after the procedure. Except for an abscess on the right elbow, no other external signs of infection, including a sensation of local heating or erythematous alterations, were found. Cultures of the abscess were obtained at the clinic. The patient was afebrile during the course, and blood culture was not performed. Laboratory tests showed an increased ESR and CRP concentration (31 mm/hour and 8.6 mg/L, respectively). Pulmonary CT showed multiple microcalcifications in the right upper lobe (similar to the results of the CT scan performed 4 months prior), suggesting stable pulmonary tuberculosis (TB). However, the initial 2 cultures of the abscess in the clinic revealed negativity. The initial treatment comprised intravenous antibiotics for gram-positive and gram-negative bacteria; however, the presentation was not controlled, and the abscess eventually deteriorated with yellow effusion. Based on the clinical presentation, the patient was diagnosed with a periprosthetic infection. Debridement was then performed without removing the prosthesis. Cultures of the wound tissues were obtained for further diagnosis. During debridement, specific manifestations of TB, such as caseous necrosis, were not observed in the tissues. Despite performing debridement and administering intravenous antibiotics, the wound failed to heal. The patient exhibited no clinical manifestations of TB, but further examination showed positive T-SPOT test results. The TB test and tuberculin purified protein derivation (PPD) test results were positive, and fortunately, Corynebacterium exhibited growth on cultures from the debridement. According to these cultures and suggestions of a bacteriologist, the antibiotic regimen was changed to isoniazid (0.3 g, po qd), rifampin (0.45 g, po qd), pyrazinamide (0.5 g, po tid), and ethambutol (0.75 g, po qd). Three weeks later, the wound healed, and the symptoms had significantly relieved. Cultures eventually grew into *M. tuberculosis*, so the anti-TB treatment was continued for 12 months. At the time of writing this case report, the elbow prosthesis has survived for more than 2 years, and no recurrent infection has been observed.

## 3 Discussion

Prosthetic joint infections (PJIs) due to TB are uncommon, and no studies with large cohorts have been reported. In a recent synthesis of the literature, TB-PJIs have been found to usually involve the hip (35 cases, 53%) or knee (27 cases, 40.9%), but they can involve any joint (shoulder, elbow, and wrist).^[4]^*M. tuberculosis* infection after TEA is extremely rare, with only a single case previously reported by Asopa and Wallace^[5]^ in which a 37-year-old man presented with swelling 2 months after undergoing elbow replacement. There are 2 hypothesized pathogenic mechanisms: local reactivation of previous TB caused by tissue trauma related to the surgery and hematogenous spread in patients with an active or latent TB infection.^[6]^ There were no prior clinical manifestations of TB in the elbow in our case; thus, the possible pathogenic mechanism is that it was obtained by hematogenous spread from a latent pulmonary TB infection.

The diagnosis of a TB infection after replacement remains a challenge for orthopedic surgeons. Due to its atypical clinical presentation, such as pain, swelling, and sinus complications, diagnosis is often delayed. Regarding diagnostic imaging, plain radiographs are not specific, and CT and magnetic resonance imaging are limited by prothesis-associated artefacts.^[7]^ Patients with an underlying immunocompromised condition, history of TB infection, or TB risk factors (such as advanced age, obesity, diabetes, autoimmune disorders, chronic steroid therapy, and immunomodulating medications) should consider the possibility of postoperative TB infection prophylaxis.^[8]^ Herein, the patient was immunosuppressed due to long-term use of tripterygium glycoside and methotrexate for RA, and pulmonary CT showed multiple microcalcifications in the right upper lobe, suggesting stable pulmonary TB and the suspicion of TB-PJI was necessary.

At present, acid-fast bacilli staining and cultures remain the gold standard for the diagnosis of TB-PJIs, but microbiological confirmation is difficult to obtain. Similar to our results, synovial fluid cultures have a low sensitivity for the diagnosis of TB-PJIs, and in most previous cases, patients were diagnosed based on periprosthetic tissue cultures.^[9]^ In addition, the growth of TB in cultures often takes a few weeks, with an average of 23.7 days.^[10]^ Results of laboratory tests can either be within normal reference ranges or show increased inflammatory indicators such as the ESR and CRP concentration. These indicators act as markers of general inflammatory activity and have low specificity. Polymerase chain reaction (PCR) assays are a suitable alternative to detect TB with the advantages of high sensitivity and specificity and rapid turnaround.^[11]^ Neogi et al reported a patient who developed a PJI 14 years after total knee arthroplasty, while cultures from synovial tissue and joint fluid revealed negativity. A TB-PJI was finally diagnosed in the patient based on PCR detecting *M. tuberculosis* in the synovial tissue.^[12]^

Currently, there are no guidelines for managing patients with TB-PJIs. The basic treatment strategy includes anti-TB medications, early diagnosis and treatment, or empiric therapy in cases wherein a diagnosis might be delayed. However, the optimal surgical strategy for treating TB-PJIs remains unknown, and there is no consensus on whether to retain the prosthesis.^[13]^ The only case of a TB-PJI after TEA reported previously was managed with a two-stage revision along with anti-TB medications for 6 months. The patient had a satisfactory functional and pain-free range of motion.^[5]^ However, Uhel et al^[14]^ reported a case series of TB-PJIs after hip and knee replacements and found that 93% (13/14) of patients with TB-PJIs who did not undergo surgery had favorable outcomes, suggesting that prolonged anti-TB treatment may be curative in a substantial proportion of cases. In our case, we decided to extend the medications to 12 months without removing the prosthesis. The patient exhibited a favorable outcome, and no recurrent infection was observed.

In developing countries, where TB remains common, the number of TEAs performed is increasing. Despite the extremely low incidence, in cases of PJIs that do not respond to conventional treatment, the suspicion of TB-PJIs should be encouraged, especially in patients with risk factors. Proper diagnosis allows for antitubercular therapy with retention of the prosthesis.

## Acknowledgments

We would like to thank Editage (www.editage.cn) for English language editing.

## Author contributions

**Writing – original draft:** Yun Guan.

**Writing – review & editing:** Zhimin Zeng.
